# RAD18 O-GlcNAcylation promotes translesion DNA synthesis and homologous recombination repair

**DOI:** 10.1038/s41419-024-06700-y

**Published:** 2024-05-08

**Authors:** Xiaolu Ma, Hui Fu, Chenyi Sun, Wei Wu, Wenya Hou, Zibin Zhou, Hui Zheng, Yifei Gong, Honglin Wu, Junying Qin, Huiqiang Lou, Jing Li, Tie-Shan Tang, Caixia Guo

**Affiliations:** 1 Key Laboratory of Organ Regeneration and Reconstruction, State Key Laboratory of Membrane Biology, Institute of Zoology, Chinese Academy of Sciences, Beijing, 100101 China; 2 Beijing Institute of Genomics, Chinese Academy of Sciences/China National Center for Bioinformation, Beijing, 100101 China; 3 College of Biomedical Engineering, Taiyuan University of Technology, Taiyuan, 030024 China; 4 University of Chinese Academy of Sciences, Chinese Academy of Sciences, Beijing, 100101 China; 5 Shenzhen University General Hospital, Guangdong Key Laboratory for Genome Stability & Disease Prevention, Shenzhen University School of Medicine, Shenzhen, Guangdong China; 6 Beijing Key Laboratory of DNA Damage Response and College of Life Sciences, Capital Normal University, Beijing, 100048 China; 7 Beijing Institute for Stem Cell and Regenerative Medicine, Beijing, 100101 China

**Keywords:** Glycosylation, Translesion synthesis

## Abstract

RAD18, an important ubiquitin E3 ligase, plays a dual role in translesion DNA synthesis (TLS) and homologous recombination (HR) repair. However, whether and how the regulatory mechanism of O-linked N-acetylglucosamine (O-GlcNAc) modification governing RAD18 and its function during these processes remains unknown. Here, we report that human RAD18, can undergo O-GlcNAcylation at Ser130/Ser164/Thr468, which is important for optimal RAD18 accumulation at DNA damage sites. Mechanistically, abrogation of RAD18 O-GlcNAcylation limits CDC7-dependent RAD18 Ser434 phosphorylation, which in turn significantly reduces damage-induced PCNA monoubiquitination, impairs Polη focus formation and enhances UV sensitivity. Moreover, the ubiquitin and RAD51C binding ability of RAD18 at DNA double-strand breaks (DSBs) is O-GlcNAcylation-dependent. O-GlcNAcylated RAD18 promotes the binding of RAD51 to damaged DNA during HR and decreases CPT hypersensitivity. Our findings demonstrate a novel role of RAD18 O-GlcNAcylation in TLS and HR regulation, establishing a new rationale to improve chemotherapeutic treatment.

## Introduction

The E3 ubiquitin ligase RAD18 coordinates translesion DNA synthesis (TLS), homologous recombination (HR) and other DNA damage response pathways to maintain genome integrity [1–6]. As a conserved and predominant DNA damage tolerance mechanism, TLS is regulated by the monoubiquitination of proliferating cell nuclear antigen (PCNA), which acts as a ring-shaped homotrimer encircling DNA as a scaffold to promote multiple specialized TLS polymerases to replicative bypass ultraviolet (UV)- and cisplatin (cis-diamminedichloroplatinum, CDDP)-induced damage [6–8]. Remarkably, RAD18 in complex with the E2 ubiquitin conjugase RAD6 is specifically required for the monoubiquitination of PCNA (mUb-PCNA) at Lys164 [6, 9]. Apart from its traditional role in TLS, RAD18 has also been shown to play an integral role in the repair of DNA double-strand breaks (DSBs) by directly binding to the recombinase RAD51C, a paralog of RAD51 and localizing it to DSBs to orchestrate HR [1]. The recruitment of RAD18 to DSBs is thought to depend on an interaction between the ubiquitin-binding zinc finger (UBZ) domain of RAD18 and ubiquitinated proteins on chromatin. Deletion of the RAD18 UBZ domain abolished the loading of RAD18 to DSB sites and therefore eliminated its role in HR repair [1, 4]. Accumulating evidence indicates that the expression level of RAD18 contributes to mutagenesis and DNA damage-based cancer therapy resistance [10–19], hinting the necessity and significance of understanding the regulatory mechanism governing RAD18 function in maintaining genome stability and tumorigenesis.

Due to the lack of PCNA-binding motifs in RAD18, several adapter factors including replication protein A, SIVA1 apoptosis-inducing factor (SIVA1), SprT-like N-terminal domain (Spartan) and Nijmegen breakage syndrome 1 (NBS1) have been reported to regulate RAD18 access to stalled replication forks and PCNA [20–24]. Specifically, RAD18 could be targeted to PCNA by TLS polymerase eta (Polη). Polη physically binds to RAD18, in turn targeting it to PCNA and stimulating PCNA monoubiquitination, a function fully dissociable from its TLS polymerase activity [3]. In addition to interactions with various DNA repair proteins, RAD18 activity also depends on post-translational modifications (PTMs), such as phosphorylation, ubiquitination, and SUMOylaltion. For instance, monoubiquitinated RAD18 is responsible for its interaction with the UBZ domain in RAD18 and the subsequent formation of the RAD18 homodimer to prevent TLS overactivation [25–27]. Moreover, cell cycle kinase CDC7-dependent Ser434 phosphorylation of RAD18 is essential for recruiting Polη to sites of UV-induced DNA damage [28]. Recently, O-Linked β-N-acetylglucosamine (O-GlcNAc) transferase (OGT) has been reported to localize to DNA lesions and promote the O-GlcNAcylation at serine and threonine residues of DNA repair factors upon UV, CDDP and ionizing radiation (IR) treatment [29–34]. Nevertheless, an enigma remains of how O-GlcNAcylation may govern RAD18 during TLS and HR repair.

In this study, we identified that RAD18 interacts with OGT and undergoes O-GlcNAcylation at Ser130/Ser164/Thr468. Although S130A/S164A/T468A (3A) mutation does not impair the binding of RAD18 to RAD6, SIVA1, Spartan or NBS1, it unexpectedly restrains CDC7-dependent RAD18 phosphorylation at Ser434, leading to reduced PCNA monoubiquitination, limited Polη focus formation and increased UV sensitivity, revealing novel crosstalk between RAD18 O-GlcNAcylation and phosphorylation during TLS. Intriguingly, 3A mutation significantly attenuates RAD18 ability of ubiquitin and RAD51C binding, rendering defects in RAD51 accumulation, HR repair and cell survival after CPT treatment. Therefore, O-GlcNAcylation plays an important role in governing RAD18 function during TLS and HR, adding a further layer of regulation to fine-tune genome stability in vivo.

## Materials and methods

### Plasmids and reagents

Human RAD18 cDNA was cloned in pEGFP-C3 (Clontech), pCMV-SFB (streptavidin-Flag-SBP) or pLVX-Mcherry vector using One Step Cloning Kit (Yeasen Biotechnology, Shanghai). Different RAD18 mutants including S130A, S164A, T468A, S434A and 3A (S130A/S164A/T468A) were constructed by site-mutated PCR amplification. His-SUMO-RAD18 plasmid and anti-Ubiquityl-PCNA (Lys164) (13439, Cell Signaling Technology) antibody were gifts from Dr Jun Huang (Zhejiang University, Hangzhou, China). Anti-Flag M2 agarose affinity gel (A2220) was purchased from Sigma (St Louis, MO). Anti-GFP nanobody agarose beads (KTSM1301) were from AlpaLife. Streptavidin sepharose beads (17511301) were from Cytiva. Antibodies sources were as follows: mouse anti-Flag (F1804, 1:1000) from Sigma (St Louis, MO), anti-RAD18 (ab17725, 1:1000) from Abcam and (H00056852-M01) from Novus Biologicals, anti-RAD18 pS434 from Dia-An Biotechnology, O-linked β-N-acetylglucosamine (O-GlcNAc, ab2739, 1:2000) and anti-RAD51 (ab133534, 1:200) from Abcam, anti-HA (902302, 1:2000) from BioLegend, anti-Myc (MMS-150R-500, 1:1000) from Covance, anti-H3.1 (P30266, 1:2000) from Abmart, anti-β-Tubulin (AbM59005-37-PU, 1:4000) from Beijing Protein Innovation (Beijing, China), anti-GFP (sc-8334, 1:500), OGT (sc-32921, 1:1000) and anti-PCNA (sc-56, 1:1000) from Santa Cruz Biotechnology, anti-H2B (2934S) from Cell Signaling Technology (CST). Alexa Fluor-conjugated secondary antibodies were from Invitrogen.

### Cell culture and reagents

Human U2OS and HEK293T cells were obtained from the American Type Culture Collection (Rockville, MD). RAD18 knockout (RAD18-/-) cells were established using TALEN as described previously [5]. These cell lines were grown in DMEM medium supplemented with 10% fetal bovine serum. All cells were grown at 37 °C in the presence of 5% CO_2_ if not specified. All cells were tested for mycoplasma contamination using the Lonza Mycoplasma kit. For transient transfection experiments cells were transfected with indicated constructs, using Vigofect (Vigorous Biotechnology) following the manufacturer’s protocols. For RNAi experiments, cells were transfected with siRNAs purchased from GenePharma (Shanghai, China) using RNAiMAX (Invitrogen) according to manufacturer’s instruction, and analyzed 72 h later. The gene-specific target sequences were as follows: RAD18 (GCAAGGACCUGCUGUUUAU), OGT (GAUUAAGCCUGUUGAAGUC; UAAUCAUUUCAAUAACUGCUUCUGC). The negative control (siNC) sequence (UUCUCCGAACGUGUCACGU) was also obtained from GenePharma. For shRNA knockdown, oligonucleotide encoding shRNA of RAD18 was synthesized by Generay (Shanghai, China). The oligonucleotides were annealed and cloned into pLKO.1 vector (Biovector) to generate shRAD18 vectors. The shRAD18 sequence is as follows: GAGATGAGGTTTCACCATGTTTTCAAGAGAAACATGGTGAAACCTCATCTC.

### Laser microirradiation and imaging

Cells were seeded in 35 mm glass-bottomed cell culture dish (Biosharp) and then cultured for 24 h before transfecting with WT or 3A GFP-RAD18 plasmids. 24 h after transfection, GFP-RAD18-expressing cells were selected for laser microirradiation by a 365-nm pulsed nitrogen ultraviolet laser. Images were captured at 10 sec intervals by DragonFly confocal imaging system and analyzed by Image J software.

### Chromatin fractions isolation

Cells were lysed in CSK-100 buffer (100 mM NaCl, 300 mM sucrose, 3 mM MgCl_2_, 10 mM PIPES pH 6.8, 1 mM EGTA, 0.2% Triton X-100) containing protease inhibitors at 4 °C for 15 min. Chromatin-associated proteins were released from the pellets by treatment with lysis buffer (50 mM HEPES pH 7.5, 50 mM NaCl, 0.05% SDS, 2 mM MgCl_2_, 10% Glycerol, 0.1% Triton X-100, 10 units of Benzonase nuclease) containing protease inhibitors at 4 °C overnight. The supernatants were separated by SDS-PAGE and detected by immunoblotting with indicated antibodies.

### Immunofluorescence

Cells were cultured on glass coverslips. Briefly, cells were treated with 0.5% Triton X-100 for 5 min before fixing in 4% paraformaldehyde. Then the cells were incubated with 5% fetal bovine serum and 1% goat serum for 1 h followed by incubation with anti-RAD51 for 1 h. After staining with secondary antibodies (Alexa Fluor 488) for 1 h, coverslips were mounted in Vectashield mounting medium (Vector Laboratories) containing the nuclear stain 4, 6-diamidino-2-phenylindole (DAPI). For quantitative analysis of RAD18 focus formation, U2OS cells transfected with GFP-RAD18 were treated with CPT (2 μM), Bleomycin (4 μg/ml) and UV (15 J m^−2^) respectively and fixed with 4% paraformaldehyde. Images were acquired using a Leica DM5000 (Leica) equipped with HCX PL S-APO 63×1.3 oil CS immersion objective (Leica). A minimum of 200 nuclei was analyzed for each treatment.

### Co-immunoprecipitation (co-IP) and Western blotting

HEK293T cells transfected with indicated plasmids were harvested and lysed with HEPES buffer (50 mM HEPES pH 7.5, 150 mM NaCl, 1 mM EDTA, 1 mM EGTA, 10% glycerol, 1% Triton X-100, 25 mM NaF, 10 μM ZnCl_2_). The whole cell lysates were immunoprecipitated with either anti-Flag M2 agarose or GFP nanobody agarose beads. RAD18 O-GlcNAcylation was confirmed by denatured immunoprecipitation (IP) as previously described [5, 30]. Briefly, cells were lysed with 1xSDS lysis buffer (50 mM Tris-HCl pH 6.8, 100 mM DTT, 2% SDS, 10% glycerol) at 95 °C for 15 min. The supernatant was diluted with HEPES buffer (1:14) followed by IP with anti-Flag M2 beads. For chromatin fractional IP, cells transfected with indicated plasmids were harvested and permeabilized by CSK-100 buffer (100 mM NaCl, 300 mM Sucrose, 3 mM MgCl_2_, 10 mM PIPES pH 6.8, 0.2% Triton X-100) at 4 °C for 15 min. The pellet was further lysed with buffer (50 mM HEPES, 50 mM NaCl, 10% glycerol, 10 μM ZnCl_2_, 2 mM MgCl_2_, 0.05% SDS, 0.1% Triton X-100) at 4 °C overnight. The supernatant was diluted with buffer (50 mM HEPES, 50 mM NaCl, 10% glycerol, 10 μM ZnCl_2_, 0.1% Triton X-100) (1:10) followed by IP with anti-Flag M2 beads. The immunoprecipitated products were separated by SDS-PAGE and detected by immunoblotting with indicated antibodies. The relative O-GlcNAcylation or phosphorylation levels of RAD18 in each sample were represented, with the O-GlcNAcylation or phosphorylation level of the control sample set to 1 (100%). The gray densities of the O-GlcNAcylation or phosphorylation signals and those of unmodified RAD18 were determined by Photoshop software (Adobe Systems Incorporated, USA).

### In vitro O-GlcNAcylation assay

The O-GlcNAcylation in vitro assay was performed as previous [30]. In brief, pET-28a-WT or 3A RAD18 (with kanamycin resistance) was co-transformed with pGEX-4T-2-OGT (with ampicillin resistance) into *E. coli* Transetta (DE3) cells. Single clones selected on ampicillin/kanamycin plate were grown at 37 °C until they reached OD_600_ = 0.6, then isopropyl β-D-1-thiogalactopyranoside (IPTG) (0.4 mM) was added and cultured at 16 °C overnight. His-tagged RAD18 was affinity-purified using Ni-NTA agarose (Qiagen) and resolved by SDS-PAGE followed by immunoblotting with antibodies against O-GlcNAc and His.

### Protein purification and pulldown assay

His-RAD18, His-RAD51C, Glutathione-S-transferase (GST) and GST-Ubb proteins were expressed in *E. coli* Transetta (DE3) cells. The cell pellets were sonicated in buffer (50 mM imidazole pH 6.8, 100 mM NaCl, 10 mM EDTA) for GST and GST-Ubb, buffer (20 mM HEPES pH 7.5, 300 mM NaCl, 1% Triton X-100, 1 mg/ml lysozyme) for RAD18 or buffer (50 mM NaH_2_PO_4_ pH 8.0, 300 mM NaCl, 10 mM imidazole pH 6.8, 2 mM ZnCl_2_, 1 mg/ml lysozyme) for RAD51C with 1 mM PMSF and 1 mM DTT. The supernatant was incubated with Glutathione Sepharose 4B beads or Ni-NTA agarose beads for 2 h, followed by washing with low salt (20 mM imidazole pH 6.8, 100 mM NaCl, 1 mM EDTA, 0.1% Triton X-100) and high salt (20 mM imidazole pH 6.8, 1000 mM NaCl, 1 mM EDTA, 0.1% Triton X-100) buffer. The bead-immobilized fusion proteins were stored in low salt buffer at 4 °C. Purified proteins were incubated with the cell lysates expressing indicated constructs in HEPES buffer (50 mM HEPES pH 7.5, 150 mM NaCl, 1 mM EDTA, 1 mM EGTA, 10% glycerol, 1% Triton X-100, 25 mM NaF, 10 mM ZnCl_2_) at 4 °C for 2 h. The reaction was terminated by boiling for 5 min in an SDS sample buffer, and the proteins were resolved by SDS-PAGE, followed by immunoblotting with indicated antibodies.

### HR reporter assay

DR-GFP U2OS cells overexpressing pLVX-Mcherry-Flag-RAD18 WT and 3A were treated with RAD18 siRNA, followed by transfecting with I-SceI endonuclease lentiviral particles. Forty-eight hours later, the cells were harvested and examined the ratio of GFP^+^Mcherry^+^ to Mcherry^+^ cells by flow cytometry.

### Clonogenic assay

Cells were seeded into 6 cm dishes (~400 cells/dish) in triplicate and treated with the indicated doses of UV irradiation or CPT for 2 h at 37 °C. After treatment, cells were further incubated in a complete medium for 7–10 days. For UV irradiation, the cells were cultured along with caffeine (0.4 mM) for 30 min prior to treatment and then incubated in complete medium supplemented with caffeine (0.4 mM) for 7–10 days. Colonies were fixed and counted. The survival of genotoxin-exposed cells was determined by relating the cloning efficiency to that of an untreated control.

### Statistical analysis

All Statistical tests were determined with a two-sided Student’s *t*-test using PRISM software (Graphpad Software Inc.) unless otherwise noted. Differences were considered significant at *P < 0.05, **P < 0.01, ***P < 0.001, and ****P < 0.0001.

## Results

### RAD18 undergoes O-GlcNAcylation at Ser130/Ser164/Thr468

**Fig. 1:**
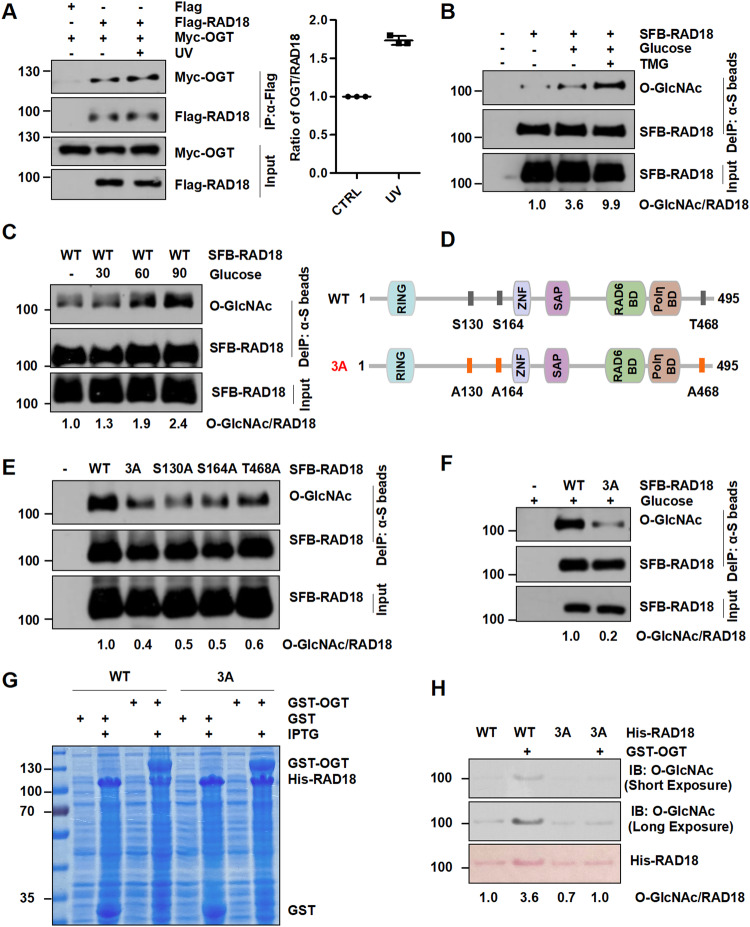
RAD18 binds OGT and is subject to O-GlcNAcylation predominantly at Ser130/Ser164/Thr468. **A** HEK293T cells expressing Flag-RAD18 and Myc-OGT were irradiated with UV (15 J m^−2^) followed by immunoprecipitation with anti-Flag M2 beads. The cell lysates were detected with anti-Myc and anti-Flag antibodies. The experiments were carried out in triplicate. **B** HEK293T cells transfected with SFB-RAD18 or empty vector were incubated with Thiamet-G (TMG) and glucose. The cell lysates were denatured and immunoprecipitated with anti-S beads followed by immunoblotting with O-GlcNAc and Flag antibodies. **C** HEK293T cells transfected with SFB-RAD18 were treated with Thiamet-G and different concentrations of glucose. The cell lysates were immunoprecipitated and analyzed as in (**B**). **D** Schematic representation of WT- and 3A-RAD18. HEK293T cells (**E**) or cells incubated with glucose and TMG (**F**) were transfected with the indicated SFB-RAD18 constructs, followed by immunoprecipitation as in (**B**). **G** Expression of His-RAD18 (WT or 3A) and GST-OGT or GST co-transformed into *E. coli* Transetta (DE3) cells after IPTG (0.4 mM) induction were examined via SDS-PAGE followed by Coomassie blue staining. **H** His-RAD18 proteins were purified and analyzed by western blot with anti-O-GlcNAc and anti-His antibodies.

As a first step to studying a possible role of O-GlcNAcylation in regulating RAD18 functions in vivo, we first investigate whether RAD18 binds to OGT. As is shown, OGT was found to bind to RAD18 in HEK293T cells, and the binding of OGT to RAD18 was substantially enhanced after UV irradiation (Fig. 1A and Supplementary Fig. 1A). To verify the O-GlcNAcylation of RAD18, we exogenously expressed SFB (streptavidin-Flag-S protein)-RAD18 in HEK293T cells and performed immunoprecipitation under a denaturing condition. A band corresponding to O-GlcNAcylated RAD18 was observed, and supplementation of glucose and Thiamet-G, the OGA inhibitor that suppresses the reversible removal of O-GlcNAc moiety from proteins, considerably increased RAD18 O-GlcNAcylation (Fig. 1B). Consistently, endogenous RAD18 undergoes O-GlcNAcylation in vivo (Supplementary Fig. 1B). Additionally, the level of RAD18 O-GlcNAcylation positively correlates with the glucose concentration in the medium (Fig. 1C).

To further determine the potential O-GlcNAcylation residue(s) in RAD18, we investigated a comprehensive O-GlcNAc modification website, namely The human O-GlcNAcome database (www.oglcnac.mcw.edu), in which most of O-GlcNAcylated sites of specific proteins were identified by mass spectrometry [35]. We found that RAD18 O-GlcNAcylation mainly occurs on three residues (Ser130, Ser164 and Thr468) [36–38]. To validate the result, we generated three single mutants (S130A, S164A or T468A) and a 3A (S130A/S164A/T468A) mutant, in which serine (S) and threonine (T) residues were mutated to alanines (A) (Fig. 1D). As shown in Fig. 1E, all RAD18 mutants, whether it was a single mutant or 3A, manifested reduced levels of O-GlcNAcylation. In the presence of Thiamet-G and glucose, the O-GlcNAcylation level of 3A-RAD18 was significantly reduced compared to that of WT-RAD18 (Fig. 1F). Sequence alignment shows that RAD18 Ser130, Ser164 and Thr468 are highly conserved in multiple organisms (Supplementary Fig. 1C). Furthermore, we purified His-tagged WT- or 3A-RAD18 co-expressing with GST-OGT or GST in *Escherichia coli* cells and found that WT but not 3A RAD18 exhibited a strong O-GlcNAcylation signal (Fig. 1G, H), supporting the notion that the majority of RAD18 O-GlcNAcylation occur within Ser130, Ser164 and Thr468 residues.

### RAD18 is recruited to DNA damage sites in an O-GlcNAcylation-dependent manner

**Fig. 2:**
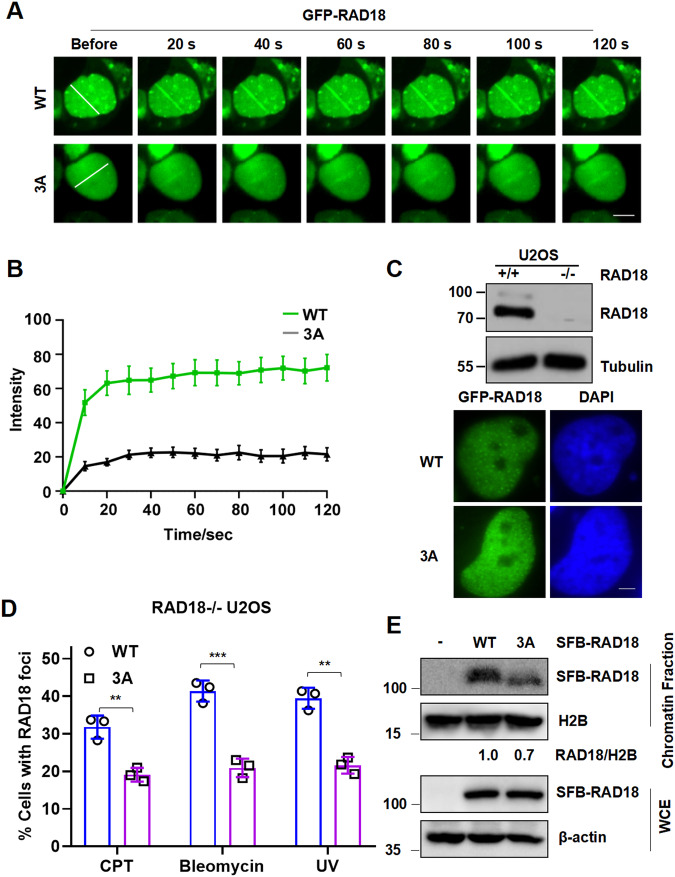
O-GlcNAclylation modulates RAD18 recruitment at DNA damage sites. **A** Representative image for the dynamic recruitment of WT or 3A GFP-RAD18 to laser-induced DSBs. Data were presented as mean ± SEM from 15 cells. Scale bar, 10 μm. **B** Quantification of the time course of WT or 3A GFP-RAD18 recruitment after laser microirradiation. **C** Representative images of GFP-RAD18 foci stained with DAPI after UV irradiation. Scale bars: 2 μm. The protein levels of RAD18 in RAD18+/+ and RAD18-/- U2OS cells were detected by immunoblotting. **D** Quantification of the percentage of RAD18-/- U2OS cells transfected with WT or 3A GFP-RAD18 constructs with more than 30 RAD18 foci after CPT, Bleomycin and UV exposure by counting at least 200 cells in each experiment. Data represent means ± SEM from three independent experiments. **E** Chromatin fractions of HEK293T cells expressing WT or 3A SFB-RAD18 were extracted followed by immunoblotting with Flag and H2B antibodies. The whole cell extract (WCE) was harvested and immunoblotted with Flag and β-actin antibodies.

Since RAD18’s function in DNA damage response (DDR) is strictly congruent with its recruitment to damaged chromatin, we speculated that O-GlcNAcylation is required for its recruitment at damage sites. To test that, we transfected WT- or 3A-RAD18 into U2OS cells and performed laser microirradiation. Remarkably, the recruitment of 3A-RAD18 to laser-induced damage sites was significantly blocked (Fig. 2A, B). Laser microirradiation induces a variety of DNA lesions at the same time, including base oxidation, UV adducts, single-strand breaks (SSBs) and DSBs. To further determine the specific effect of RAD18 O-GlcNAcylation on type(s) of DNA lesions, we treated WT- or 3A-RAD18 expressing RAD18 knockout (RAD18-/-) U2OS cells with camptothecin (CPT), bleomycin and UV. Similar observations were made in 3A-RAD18 expressing cells (Fig. 2C, D), suggesting that abolishing RAD18 O-GlcNAcylation by 3A affected RAD18 focus formation at DNA damage sites. In line with it, chromatin fractionation results revealed that 3A mutation remarkably impaired the binding of RAD18 with chromatin (Fig. 2E).

### RAD18 O-GlcNAcylation promotes PCNA monoubiquitination and cell survival after UV irradiation

**Fig. 3:**
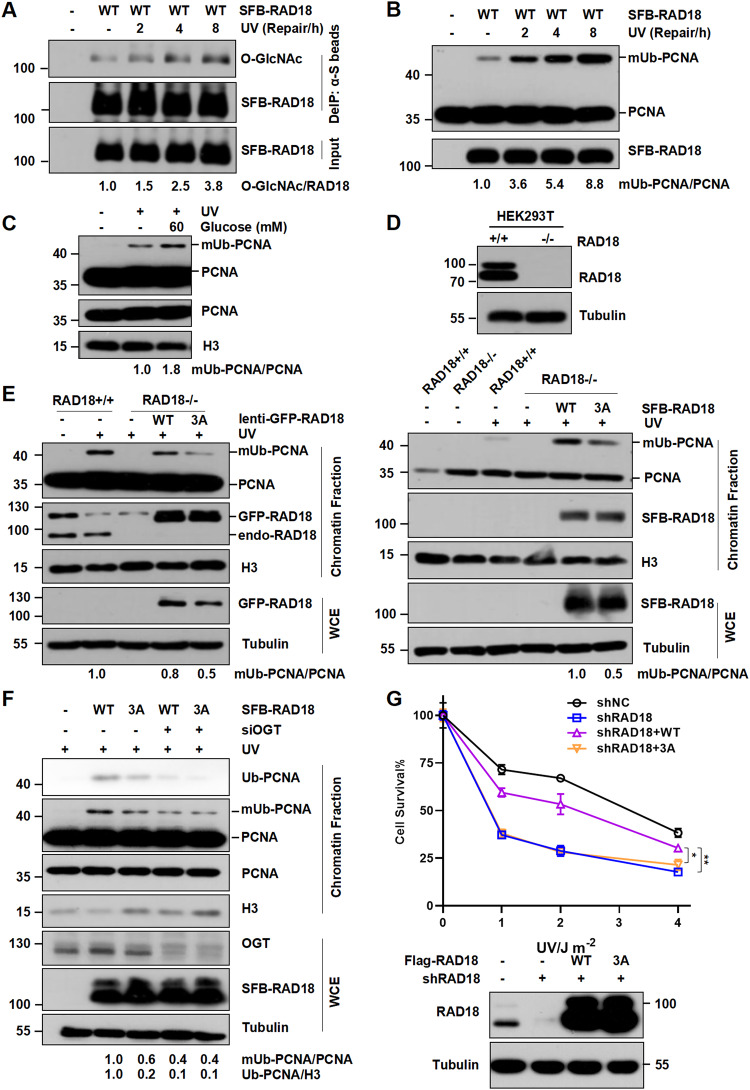
RAD18 3A mutation impairs PCNA monoubiquitination and cell survival after UV irradiation. **A** HEK293T cells transfected with SFB-RAD18 were irradiated with UV (15 J m^−2^) and harvested at different time points later. The cell lysates were denatured and immunoprecipitated with anti-S beads followed by immunoblotting with O-GlcNAc and Flag antibodies. Chromatin fractions of HEK293T cells transfected with SFB-RAD18 (**B**) or incubated with glucose or not (**C**) were extracted after UV (15 J m^−2^) irradiation followed by immunoblotting with indicated antibodies. RAD18 O-GlcNAcylation promotes PCNA monoubiquitination. RAD18+/+ or RAD18-/- HEK293T (**D**) and U2OS (**E**) or OGT-depleted RAD18-/- HEK293T (**F**) cells were transfected with WT or 3A RAD18, and irradiated with UV (15 J m^−2^). The chromatin fractions were harvested and immunoblotted using indicated antibodies. **G** Clonogenic survival assays in RAD18-knockdown U2OS cells transfected with empty vector, WT or 3A Flag-RAD18 after UV irradiation. Cells were irradiated with indicated doses of UV and further incubated in medium supplemented with 0.4 mM caffeine for 7–10 days. Surviving fraction was expressed as a percentage of mock-treated cells. The representative curve is shown. Error bar: s.d., *n* = 3. The protein levels of RAD18 were detected by immunoblotting.

To examine the biological function of O-GlcNAc modification of RAD18 in TLS, a denatured immunoprecipitation was carried out using HEK293T cells expressing SFB-RAD18. We found that the level of RAD18 O-GlcNAcylation was dramatically increased and exhibited a dynamic change after UV, which is in line with UV-induced mUb-PCNA (Fig. 3A, B), suggesting that O-GlcNAc modification of RAD18 plays a potential role in cellular response to UV exposure. For decades, it has been known that RAD18 is responsible of mUb-PCNA and TLS polymerase recruitment during TLS. Since UV-induced mUb-PCNA was increased after glucose treatment (Fig. 3C), we then sought to examine the possible role of RAD18 O-GlcNAcylation in promoting PCNA monoubiquitination. Intriguingly, UV-induced PCNA monoubiquitination was remarkably abrogated in RAD18-/- HEK293T and U2OS cells ectopically expressing 3A-RAD18 in the presence or absence of glucose compared to supplementing with WT-RAD18 (Fig. 3D, E and Supplementary Fig. 2A). Similar results were obtained when comparing RAD18-/- U2OS clones complemented with WT or 3A RAD18 with levels close to endogenous RAD18 (Supplementary Fig. 2B). To exclude the possibility that other potential types of modification at these three sites instead of O-GlcNAcylation promoting RAD18-mediated mUb-PCNA, OGT depletion was further included. Although complementing with WT-RAD18 could significantly stimulate UV-induced mUb-PCNA, loss of OGT downregulated the stimulatory effect, with WT- and 3A-RAD18 expressing cells exhibited similar extent of low mUb-PCNA levels upon OGT depletion (Fig. 3F). In line with this notion, 3A-RAD18 significantly abrogated mUb-PCNA post-CDDP, which induces the damage that could be bypassed by TLS (Supplementary Fig. 2C). Collectively, these data suggest that RAD18 O-GlcNAcylation promotes PCNA monoubiquitination.

To further explore the physiological relevance of RAD18 O-GlcNAcylation, we determined whether 3A mutants would be defective in restoring cell survival after UV irradiation. Expectedly, WT-RAD18, but not 3A-RAD18 partially restored the UV hypersensitivity in RAD18-knockdown U2OS cells (Fig. 3G). Hence, O-GlcNAcylation at Ser130/Ser164/Thr468 is required for the TLS function of RAD18.

### 3A mutation impairs CDC7-mediated RAD18 phosphorylation at Ser434

**Fig. 4:**
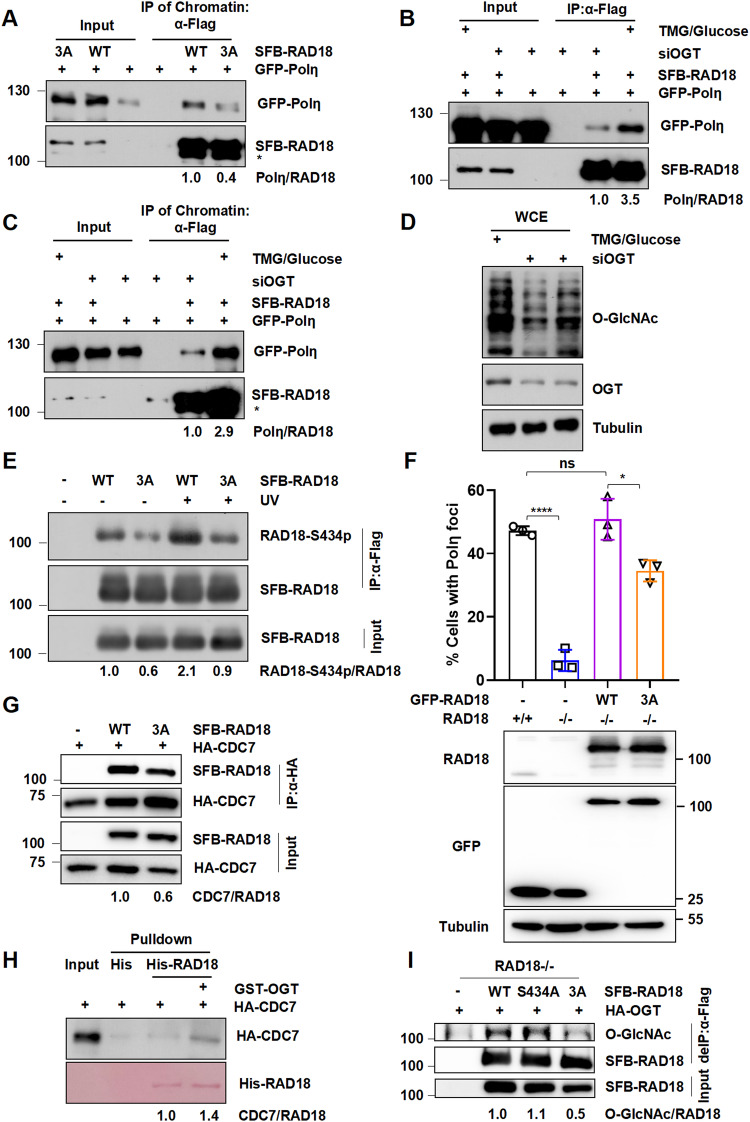
O-GlcNAclylation promotes CDC7-mediated RAD18 phosphorylation at Ser434. **A** WT or 3A SFB-RAD18 and GFP-Polη co-transfected HEK293T cells were treated with glucose (60 mM) and TMG (5 μM). Chromatin fraction was immunoprecipitated followed by immunoblotting with GFP and Flag antibodies after UV (15 J m^−2^) irradiation. *non-specific band. OGT knockdown HEK293T cells were transfected with WT or 3A SFB-RAD18 and GFP-Polη, followed by incubation with glucose (60 mM) and TMG (5 μM) or not. The whole cell lysates (**B**) and triton-insoluble fraction (**C**) were immunoprecipitated and immunoblotted with indicated antibodies post UV (15 J m^−2^) exposure. *non-specific band. **D** OGT and O-GlcNAcylation levels in (**B,** **C**). **E** SFB-RAD18 (WT or 3A) were transfected into RAD18-/- HEK293T cells followed by UV (15 J m^−2^) irradiation. The lysates were immunoprecipitated and analyzed via western blot by RAD18-S434p and Flag antibodies. **F** Flag-Polη was transfected into WT or 3A GFP-RAD18-complemented RAD18-depleted U2OS cells followed by UV (15 J m^−2^) irradiation. Quantification of the percentage of GFP-RAD18-positive cells with more than 30 Polη foci after UV (15 J m^−2^) exposure by counting at least 200 cells in each experiment. Data represent means ± SEM from three independent experiments. The protein levels of RAD18 were detected by immunoblotting. **G** WT or 3A SFB-RAD18 and HA-CDC7 were transfected into HEK293T cells. The lysates were immunoprecipitated using anti-HA beads and analyzed via western blot using Flag and HA antibodies. **H** His-RAD18 co-expressed with GST or GST-OGT was incubated with HEK293T cell lysates expressing HA-CDC7. The bound proteins were resolved by SDS-PAGE and analyzed by immunoblotting with HA antibody and staining with Ponceau S. **I** HEK293T cells transfected with HA-OGT and SFB-RAD18 (WT, S434A or 3A) or empty vector were lysed in a denatured condition and immunoprecipitated with anti-Flag beads followed by immunoblotting with O-GlcNAc and Flag antibodies.

To understand the mechanism by which the 3A mutation impairs mUb-PCNA, we first investigated the possibility that O-GlcNAcylation could be essential for the interaction between RAD18 and several core adapter proteins that facilitate RAD18 binding with PCNA. Of note, 3A mutation caused an apparent decrease in the association of RAD18 with Polη, but not RAD6, SIVA1, Spartan or NBS1, in the presence or absence of UV treatment (Fig. 4A and Supplementary Fig. 3). Furthermore, knockdown of OGT and glucose starvation considerably decreased RAD18 interaction with Polη (Fig. 4B–D).

Given that the non-catalytic role for Polη in targeting RAD18 to PCNA and stimulating PCNA monoubiquitination is dependent on CDC7-dependent RAD18 phosphorylation at Ser434, it is therefore tempting to speculate that O-GlcNAcylation is likely required for timely RAD18 phosphorylation and thereby CDC7-dependent recruitment to stalled replication forks in TLS. Decreased levels of RAD18 Ser434 phosphorylation were confirmed not only in RAD18 S434A mutants, but also in WT-RAD18 with phosphatase (PPase) treatment (Supplementary Fig. 4A, B), hinting the phospho-specificity of the RAD18-pSer434 antibody. As expected, ablation of RAD18 O-GlcNAcylation at Ser130/Ser164/Thr468 significantly decreased its phosphorylation at Ser434 in the presence or absence of UV irradiation (Fig. 4E), in agreement with the restrained recruitment of 3A-RAD18 to UV-induced damage sites. Since the interaction between RAD18 and Polη is necessary for the redistribution of Polη to sites of replication fork stalling, we further examined the role of RAD18 O-GlcNAcylation in Polη focus formation. RAD18-depleted U2OS cells only manifested scanty Polη signals which is in line with the previous study [39]. Compared to WT-RAD18, 3A-RAD18 caused a significant reduction in the percentage of Polη foci-positive cells post-UV irradiation (Fig. 4F and Supplementary Fig. 4C).

To further determine why WT-RAD18 is preferentially phosphorylated instead of 3A-RAD18, we performed co-IP experiments and found that 3A mutations significantly decreased the interaction between RAD18 and CDC7 (Fig. 4G). Similar observations were made using purified WT or 3A His-RAD18 to pull down CDC7 in vitro (Fig. 4H). Therefore, the diminished Ser434 phosphorylation of RAD18 O-GlcNAc mutants is likely due to its reduced association with the kinase CDC7. Notably, unlike 3A mutants, Ser434A mutations did not impair RAD18 O-GlcNAcylation in RAD18-/- HEK293T cells (Fig. 4I). Taken together, these results demonstrated that the 3A mutation limits RAD18 phosphorylation at Ser434 by reducing the binding of RAD18 to CDC7, thereby impairing RAD18 recruitment to stalled replication forks during TLS.

### O-GlcNAcylation promotes the binding of RAD18 to ubiquitin and RAD51C

**Fig. 5:**
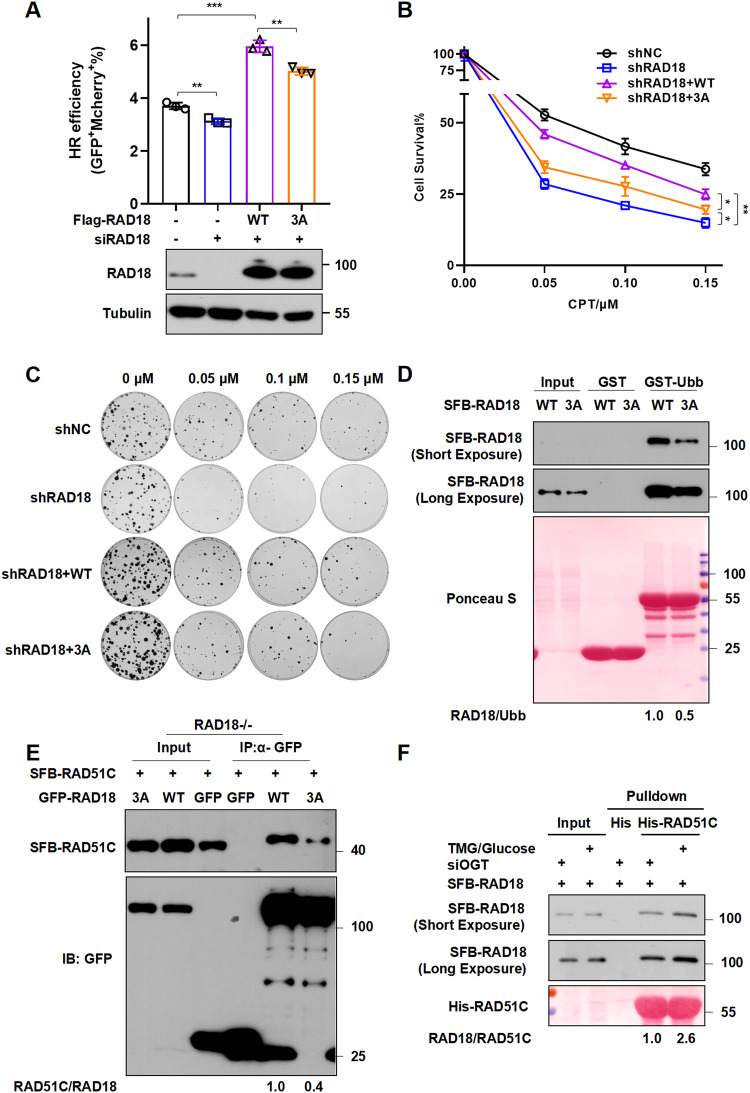
RAD18 O-GlcNAcylation is essential for its binding with both ubiquitin and RAD51C. **A** RAD18 O-GlcNAcylation promotes HR repair. DR-GFP U2OS cells overexpressing Mcherry empty vector, WT or 3A Mcherry-Flag-RAD18 were treated with siRAD18 oligo followed by I-SceI endonuclease transfection. HR repair efficiency (GFP^+^Mcherry^+^%) was analyzed by FACS. The lower panels show immunoblots indicating the RAD18 levels in different conditions. **B** RAD18 O-GlcNAcylation promotes cell survival post-CPT treatment. Clonogenic survival assays in RAD18-knockdown U2OS cells transfected with empty vector, WT or 3A Flag-RAD18 after CPT treatment. Cells were treated with indicated doses of CPT and further incubated for 7–10 days. Surviving fraction was expressed as a percentage of mock-treated cells. The representative curve is shown. Error bar: s.d., *n* = 3. **C** Representative images for colony assay in (**B**). **D** 3A mutation inhibits the binding ability of RAD18 with ubiquitin. Purified GST and GST-Ubb were incubated with HEK293T cell lysates expressing SFB-RAD18 (WT or 3A) followed by immunoblotting with Flag antibody. Ponceau staining shows the amounts of GST and GST-Ubb used in the pulldown assay. **E** RAD18-depleted HEK293T cells were transfected with WT or 3A GFP-RAD18 and SFB-RAD51C. The cell lysates were denatured followed by immunoprecipitation with anti-GFP agarose. The immunoprecipitates were immunoblotted with Flag and GFP antibodies. **F** Purified His-RAD51C was incubated with OGT knockdown HEK293T cell lysates expressing SFB-RAD18 in incubation with glucose (60 mM) and TMG (5 μM) or not. The bound proteins were analyzed as in (**D**).

RAD18 is believed to bind ubiquitin chains and contribute to the RAD51C retention at DSBs, but the regulatory mechanism of its non-canonical TLS-independent role is not clear. Since the recruitment of 3A-RAD18 to CPT-induced damage sites was significantly decreased (Fig. 2D), we speculated that O-GlcNAcylation regulates RAD18 function during HR. To directly measure the effect of O-GlcNAcylated RAD18 on HR activity, we complemented RAD18-knockdown DR-GFP U2OS cells with either WT-RAD18 or 3A-RAD18 followed by HR reporter assay. Inspiringly, compared to WT-RAD18, 3A-RAD18 reconstituted cells manifested a significant reduction of HR repair efficiency (Fig. 5A and Supplementary Fig. 5A). Moreover, 3A-RAD18 mutant failed to restore the cell survival following CPT treatment in RAD18-knockdown U2OS cells to the extent when reconstitution with WT-RAD18 (Fig. 5B, C).

**Fig. 6:**
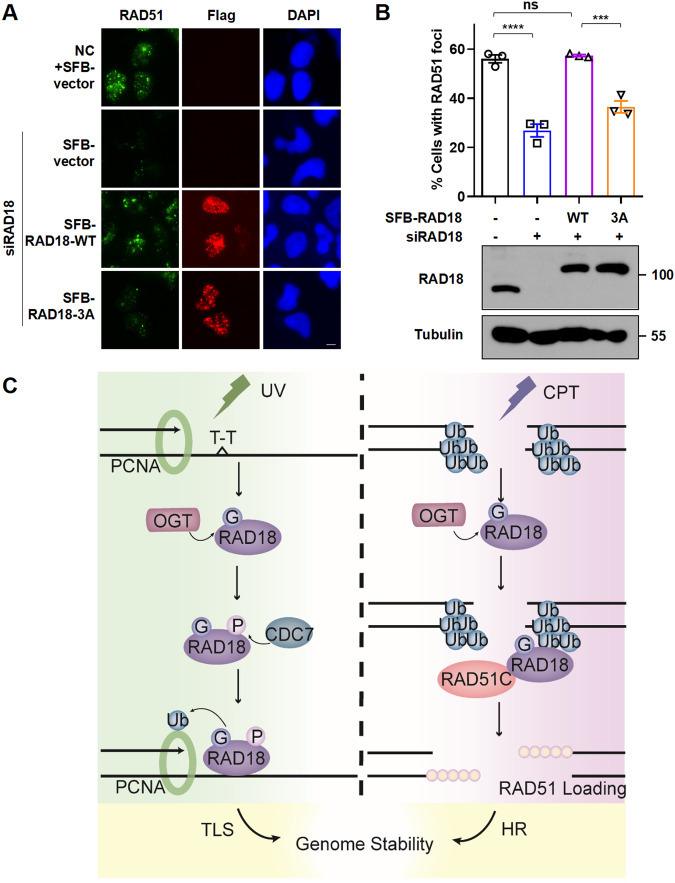
RAD18 O-GlcNAcylation facilitates RAD51 focus formation at DSBs. **A** WT or 3A SFB-RAD18 were transfected into siRAD18-treated U2OS cells followed by CPT treatment. Representative images of cells expressing RAD51 foci and SFB-RAD18 were shown. Scale bars: 5 μm. **B** The percentage of SFB-RAD18-positive cells with more than 10 RAD51 foci was determined. Data represent means ± SEM from three independent experiments by counting at least 200 cells. The protein levels of RAD18 were detected by immunoblotting. **C** Proposed model depicting the role of RAD18 O-GlcNAcylation in mediating TLS and HR pathways. Upon UV irradiation or CPT exposure, RAD18 undergoes O-GlcNAcylation by OGT. O-GlcNAcylated RAD18 promotes CDC7-dependent phosphorylation at Ser434 and further enhances PCNA monoubiquitination in TLS. O-GlcNAcylation also promotes the binding ability of RAD18 and ubiquitin chain at CPT-induced DSB sites, and further recruits RAD51C facilitating RAD51 loading during HR repair.

To further explore how RAD18 O-GlcNAcylation regulates RAD18 accumulation at DSBs and HR repair, we first performed in vitro pulldown assay using ubiquitin B-glutathione S-transferase fusion protein (GST-Ubb) and found that the association between RAD18 and ubiquitin B was significantly reduced when WT-RAD18 was mutated to 3A (Fig. 5D), in accordance with the impaired ability of 3A-RAD18 to localize to CPT- and bleomycin-induced damage sites in vivo (Fig. 2D). Given that RAD18 functions as an adapter to facilitate HR via a direct interaction with RAD51C, we next confirmed the interaction between RAD18 and RAD51C in RAD18-/- HEK293T cells co-transfecting WT or 3A GFP-RAD18 with SFB-RAD51C. As shown in Fig. 5E, RAD51C interacted with WT-RAD18, but weakly with 3A-RAD18. In vitro pulldown assays clearly demonstrated that O-GlcNAcylation of RAD18 increased association with RAD51C (Fig. 5F). Consistently, depletion of RAD18 impaired the assembly of RAD51 at CPT-induced DSB sites (Supplementary Fig. 5B), and re-introduction of WT-RAD18 but not 3A mutant restored RAD51 focus formation defect in RAD18-depleted cells (Fig. 6A, B). Taken together, RAD18 O-GlcNAcylation promotes its association with both ubiquitin and RAD51C, thereby facilitating RAD18 accumulation at DSBs and HR repair.

## Discussion

RAD18, which is typically overexpressed in cancer cells [13, 40–43], is proven to impact genome maintenance and tumorigenesis via facilitating mutagenic TLS as well as error-free HR [1, 6, 8, 9]. RAD18-mediated TLS is also potentially responsible for the spontaneous and acquired resistance of cisplatin therapy in cancer cells [44, 45]. Therefore, fine-tune of RAD18 function might be a promising approach for sensitizing cancer cells to genotoxic therapeutic agents. Protein O-GlcNAcylation is emerging as an important and abundant form of covalent modifications that regulate DNA damage repair, and many DDR-associated proteins under O-GlcNAcylation upon DNA damage, including Polη, CtIP and RAD52 [29–31, 46]. However, the role of RAD18 O-GlcNAcylation still remains unexplored. In this study, we demonstrated an augmentation of RAD18 O-GlcNAcylation after UV and CPT treatment. Inactivation of O-GlcNAcylation by 3A mutation limits the accessibility of RAD18 to chromatin, in turn impairs RAD18 focus formation and sensitizes cells to UV and CPT. Since RAD18 facilitates TLS and HR repair via distinct mechanisms, we demonstrated the role of RAD18 O-GlcNAcylation at Ser130/Ser164/Thr468 in promoting UV-induced PCNA monoubiquitination and CPT-induced RAD51 loading to maintain genome stability (Fig. 6C). Mechanistically, upon UV irradiation, O-GlcNAcylated RAD18 promotes CDC7-dependent phosphorylation at Ser434, acting with the E2 conjugating enzyme RAD6 to promote PCNA monoubiquitination at stalled replication forks. On the other hand, O-GlcNAcylated RAD18 recognizes the ubiquitin chain at DSB sites and further recruits RAD51C, facilitating RAD51 loading during CPT-induced HR repair.

Extensive crosstalk has been demonstrated between O-GlcNAcylation and other PTMs. For instance, O-GlcNAcylation at Ser149 of p53 inhibits Thr155 phosphorylation, thereby stabilizing p53 by suppressing ubiquitin-dependent proteolysis [47]. It has also been reported that abrogation of Polη Thr457 O-GlcNAcylation reduces its polyubiquitination at Lys462, leading to a delayed p97-dependent removal of Polη from replication forks and significantly enhanced UV-induced mutagenesis [30]. CDC7-mediated RAD18 phosphorylation at Ser434 directs Polη to sites of stalled replication [28]. Herein, we found inactivation of RAD18 O-GlcNAcylation inhibits its association with CDC7, leading to a distinct reduction in the level of PCNA monoubiquitination. Although S434A mutation does not impair RAD18 O-GlcNAcylation at Ser130/Ser164/Thr468, the hierarchical modification of RAD18 O-GlcNAcylation and phosphorylation need to be further determined. Therefore, our results provide further evidence for the role of RAD18 O-GlcNAcylation in TLS through a crosslink between CDC7-mediated phosphorylation. Recently, RAD18 SUMOylation, phosphorylation and ubiquitination were identified to participate in DNA damage repair. Further studies are necessary to determine how these different PTMs interplay to collaboratively regulate RAD18 function in vivo.

Given that O-GlcNAcylation is involved in the progression of multiple tumors [48–50], exploration the underlying mechanisms will be helpful to develop novel specific therapeutic target for cancer treatment. It is known that OGT and O-GlcNAcylation can be enriched at the sites of DNA damage [51]. Based on our result that mutation of the major RAD18 O-GlcNAcylation residues sensitizes cell to CPT exposure, it is reasonable to speculate that targeting RAD18 O-GlcNAcylation in cancer cells may have potential therapeutic benefits. Since the E3 ligase activity of RAD18 is not required for RAD18-mediated HR repair, it will be of great value to examine whether RAD18 O-GlcNAcylation directly alters the structure of RAD18, delineating the dynamic association/dissociation of RAD18 with ubiquitin and RAD51C at sites of damage.

Collectively, our results reveal that O-GlcNAcylation at Ser130/Ser164/Thr468 in RAD18 promotes its timely recruitment at damaged DNA sites, and subsequent PCNA monoubiquitination and RAD51 loading respectively, adding a further layer of regulation that controls TLS and HR in vivo.

### Supplementary information

Supplementary file

Original Western Blots

## Data Availability

All data supporting the findings of this study are available from the corresponding authors upon request. The full-length uncropped original western blots in the manuscript are uploaded as a single “Supplemental Material” file.
