# Supplementary material for: RAD18 O-GlcNAcylation promotes translesion DNA synthesis and homologous recombination repair
Source: Cell Death Dis. 2024 May 8;15(5):321. doi: 10.1038/s41419-024-06700-y (PMC11078974; doi:10.1038/s41419-024-06700-y)
Supplement: Supplementary file 1 — Supplementary file [file 41419_2024_6700_MOESM1_ESM.pdf]

Supplementary Information

RAD18 O-GlcNAcylation promotes translesion DNA synthesis and homologous recombination repair

Xiaolu Ma[1,2,3#], Hui Fu[2,4#], Chenyi Sun[2#], Wei Wu[2#], Wenya Hou[5#], Zibin Zhou[6], Hui Zheng[2,4], Yifei Gong[2,4], Honglin Wu[1,4], Junying Qin[2], Huiqiang Lou[5], Jing Li[6*], Tie-Shan Tang[1,4,7*], Caixia Guo[2,4*]

Figure S1. RAD18 O-GlcNAcylation mainly occurs on three conserved residues.

Figure S2. RAD18 O-GlcNAclylation promotes PCNA monoubiquitination.

Figure S3. 3A mutation has no effect on the association of RAD18 with RAD6, SIVA1, Spartan and NBS1.

Figure S4. Verification of the phospho-specificity of RAD18-pSer434 antibody.

Figure S5. 3A mutation impairs HR repair efficiency and RAD51 focus formation.

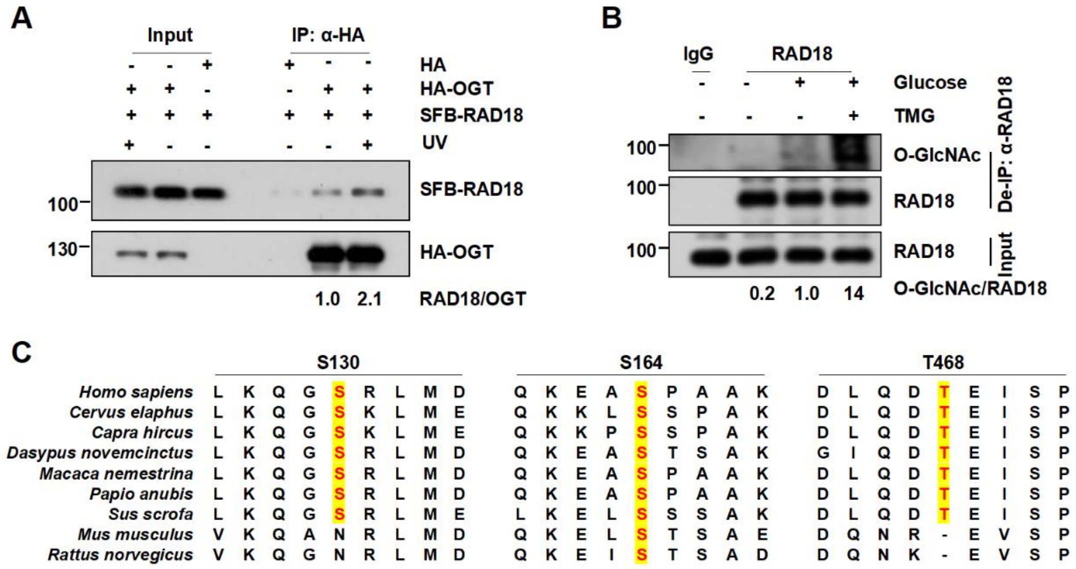

Figure S1. RAD18 O-GlcNAcylation mainly occurs on three conserved residues. A HEK293T cells expressing SFB-RAD18 and HA-OGT or HA-vector were irradiated with UV (15 J m^{-2}) followed by immunoprecipitation with anti-HA agarose beads and immunoblot with anti-HA or anti-Flag antibodies. **B** HEK293T cells were incubated with Thiamet-G (TMG) and glucose or not. The cell lysates were denatured and immunoprecipitated with anti-RAD18 antibody followed by immunoblotting with O-GlcNAc and anti-RAD18 antibodies. **C** Sequence alignment of Ser130/Ser164/Thr468 in RAD18 from different species.

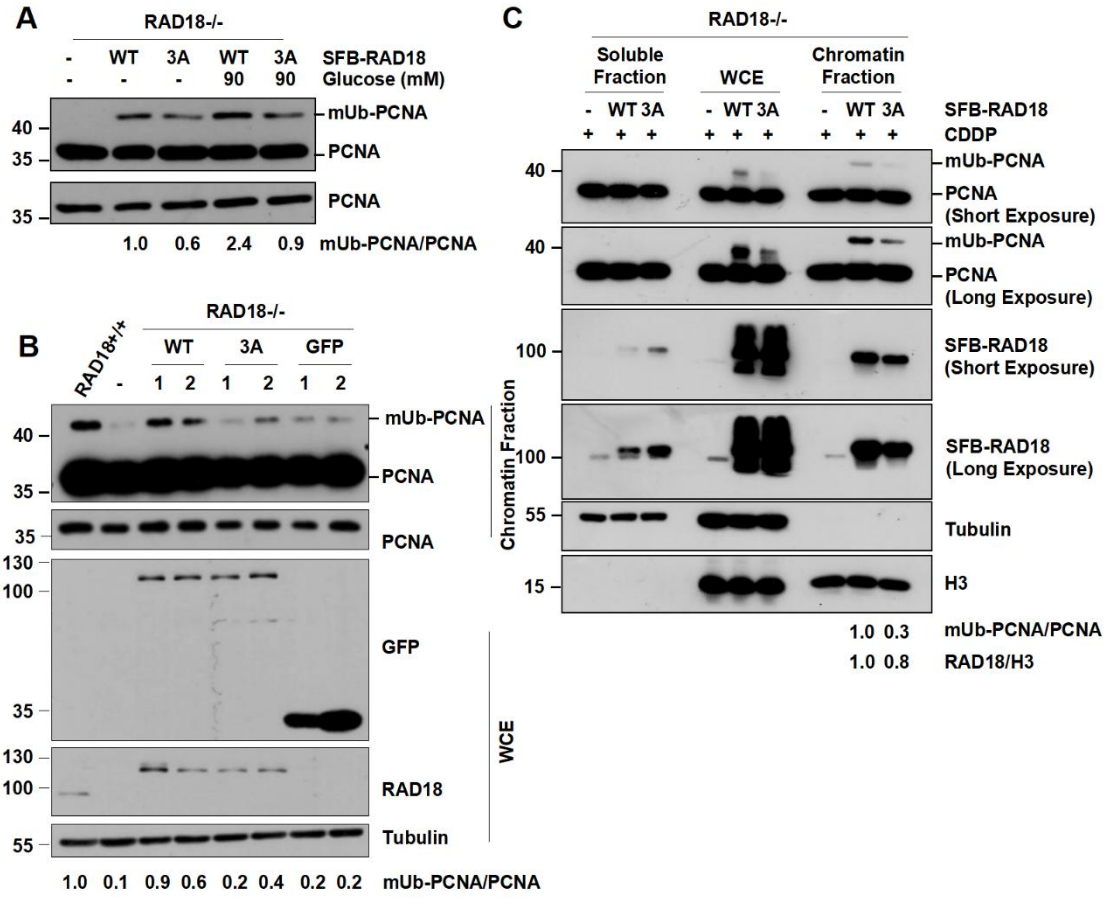

Figure S2. RAD18 O-GlcNAclylation promotes PCNA monoubiquitination. A RAD18-depleted HEK293T cells expressing WT- or 3A-RAD18 were treated with glucose. The cells were irradiated with UV (15 J m^{-2}) and the chromatin fractions were harvested and immunoblotted using indicated antibodies. **B** RAD18-/- U2OS cells were complemented with WT or 3A RAD18 through lentivirus infection to generate stable clones. The clones were irradiated with UV (15 J m^{-2}). The chromatin fractions and whole cell extracts (WCE) were harvested followed by immunoblotting using indicated antibodies. **C** RAD18-/- HEK293T cells complemented with WT- or 3A-RAD18 were treated with CDDP. Chromatin fractions, Soluble fractions or WCE were harvested and analyzed with the indicated antibodies.

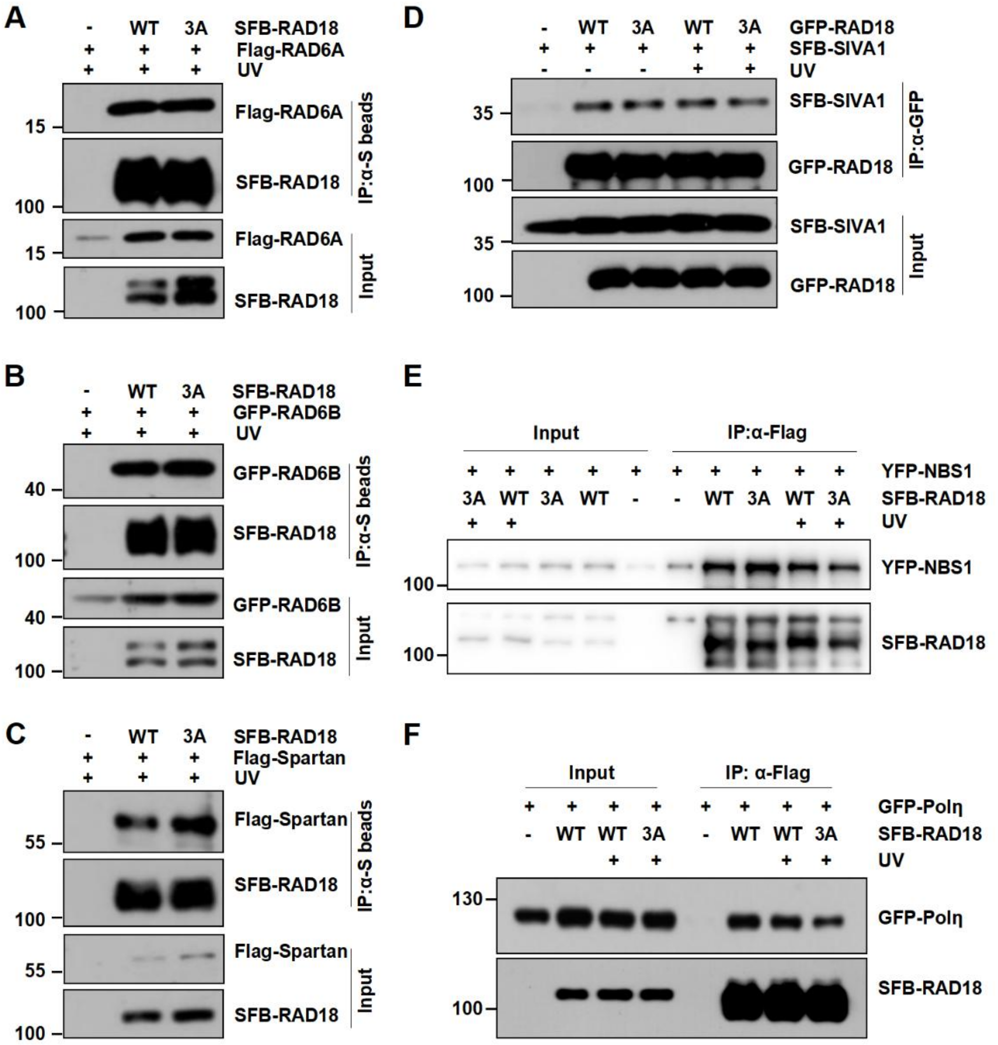

Figure S3. 3A mutation has no effect on the association of RAD18 with RAD6, SIVA1, Spartan and NBS1. A-E HEK293T cells overexpressing WT or 3A SFB-RAD18 were transfected with Flag-RAD6A (**A**), GFP-RAD6B (**B**), Flag-Spartan (**C**), SFB-SIVA1 (**D**), YFP-NBS1 (**E**) and GFP-Polη (**F**). The cell lysates were immunoprecipitated followed by immunoblotting with indicated antibodies after UV (15 J m^{-2}) irradiation.

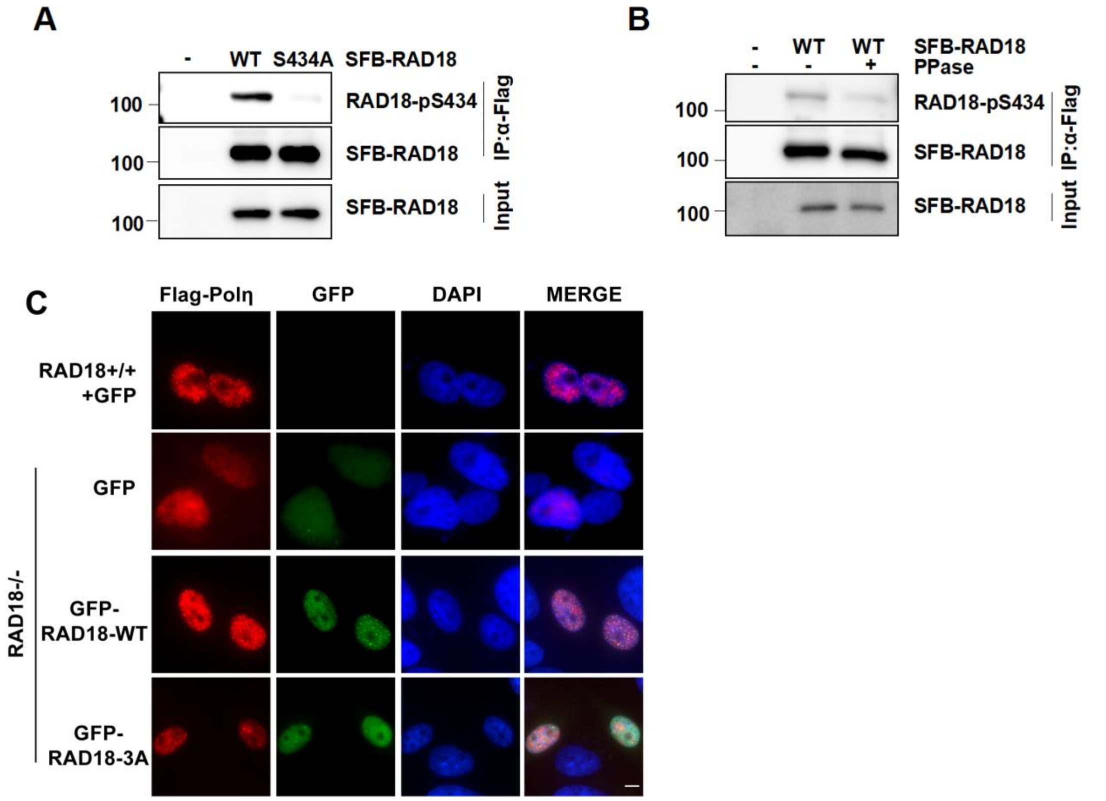

Figure S4. Verification of the phospho-specificity of RAD18-pSer434 antibody. A HEK293T cells overexpressing WT or S434A SFB-RAD18 were harvested and immunoprecipitated with anti-Flag M2 beads. RAD18 and its Ser434 phosphorylation levels were detected by western blotting. **B** SFB-RAD18 were transfected in HEK293T cells followed by PPase treatment. The lysates were immunoprecipitated and analyzed as in (**A**). **C** Flag-Polη was transfected into WT or 3A GFP-RAD18-complemented RAD18-/- U2OS cells followed by UV (15 J m^{-2}) irradiation. Representative images of cells expressing Flag-Polη foci and GFP-RAD18 are shown. Scale bars: 5 μm.

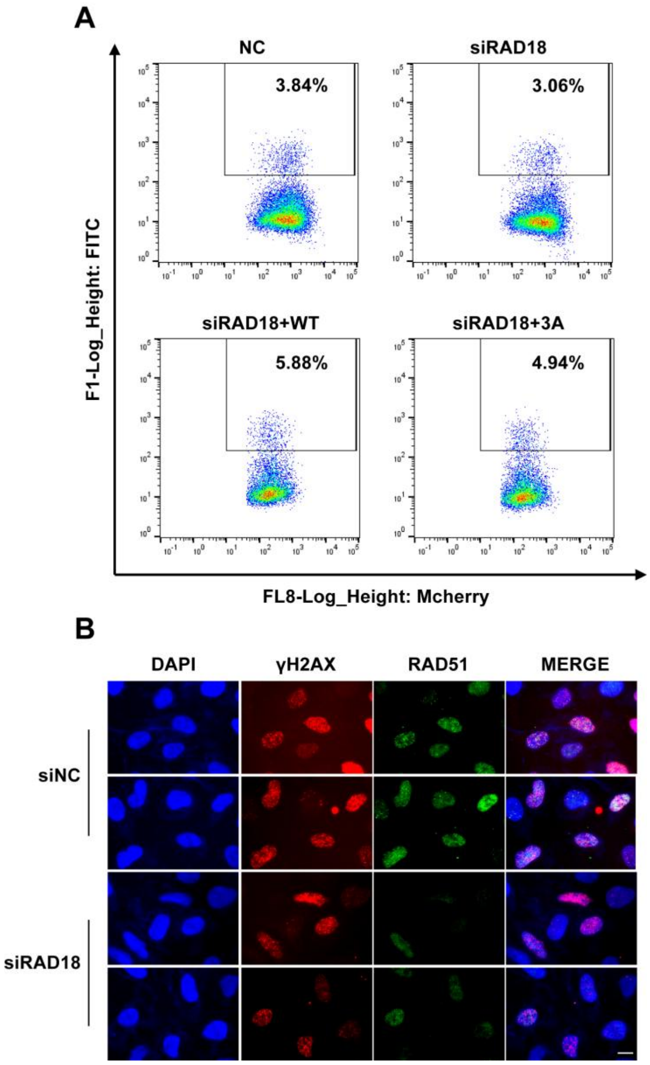

Figure S5. 3A mutation impairs HR repair efficiency and RAD51 focus formation. A Percentages of GFP- and mCherry-positive cells were examined by flow cytometry. **B** SiRAD18-treated U2OS cells were treated with CPT. Representative images of cells expressing RAD51 foci and γH2AX was shown. Scale bars: 5 μm.
