# Supplementary material for: RAD18 O-GlcNAcylation promotes translesion DNA synthesis and homologous recombination repair
Source: Cell Death Dis. 2024 May 8;15(5):321. doi: 10.1038/s41419-024-06700-y (PMC11078974; doi:10.1038/s41419-024-06700-y)

Original Western blots:

Figure 1A

Myc-OGT (IP)

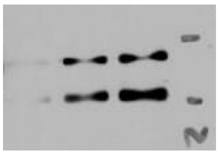

Myc-OGT (Input)

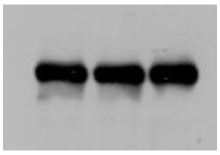

Flag-RAD18 (IP)

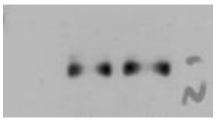

Flag-RAD18 (Input)

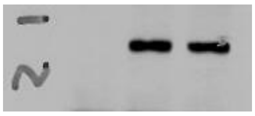

Figure 1B

O-GlcNAc

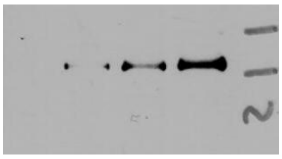

SFB-RAD18 (Input)

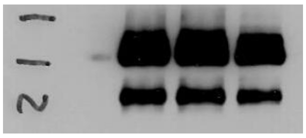

SFB-RAD18 (IP)

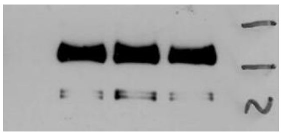

Figure 1C

O-GlcNAc

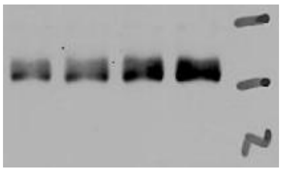

SFB-RAD18 (Input)

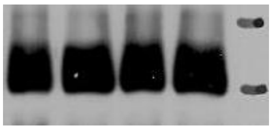

SFB-RAD18 (IP)

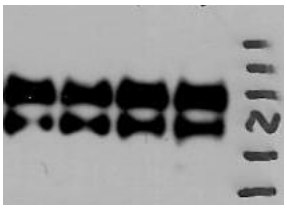

Figure 1E

O-GlcNAc

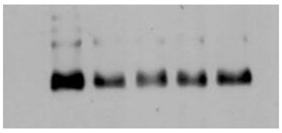

SFB-RAD18 (Input)

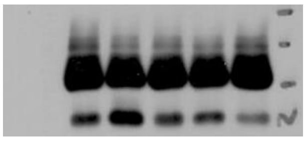

SFB-RAD18 (IP)

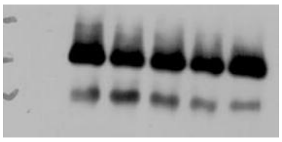

Figure 1F

O-GlcNAc

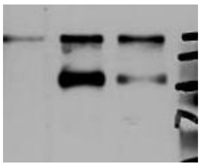

SFB-RAD18 (Input)

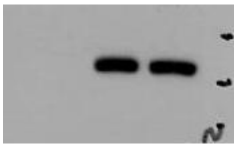

SFB-RAD18 (IP)

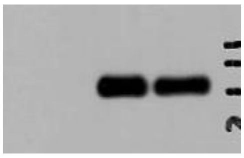

Figure 1H

O-GlcNAc (short Exposure)

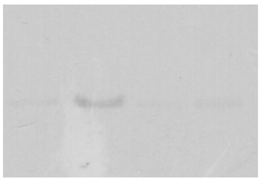

O-GlcNAc (Long Exposure)

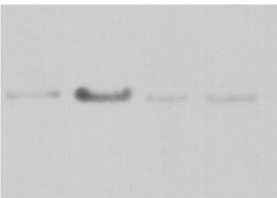

His-RAD18

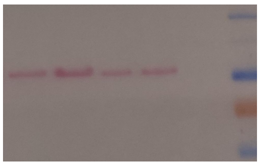

Figure 2C

RAD18

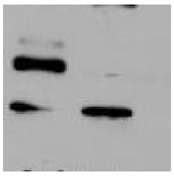

Tubulin

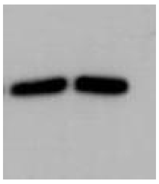

Figure 2E

SFB-RAD18 (Chromatin Fraction)

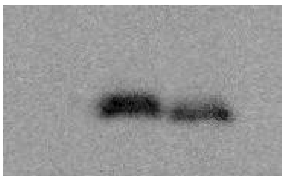

H2B

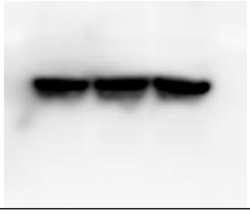

SFB-RAD18 (WCE)

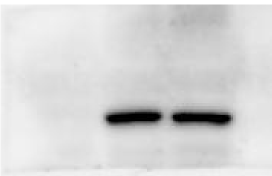

β-actin

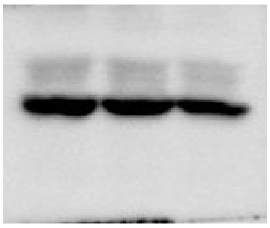

Figure 3A

O-GlcNAc

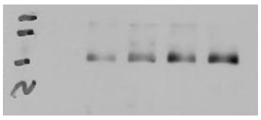

SFB-RAD18 (Input)

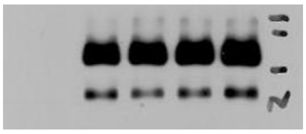

SFB-RAD18 (IP)

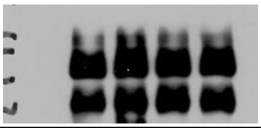

Figure 3B

PCNA

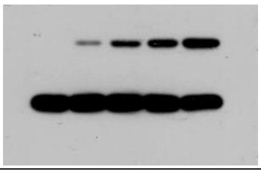

SFB-RAD18

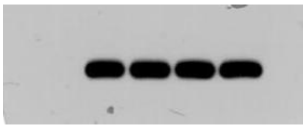

Figure 3C

mUb-PCNA

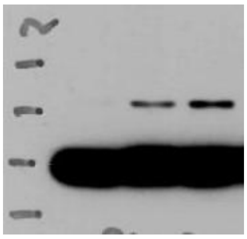

PCNA

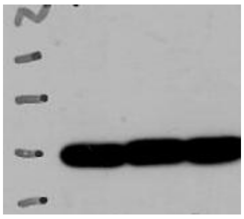

H3

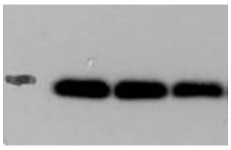

Figure 3D

PCNA (Chromatin Fraction)

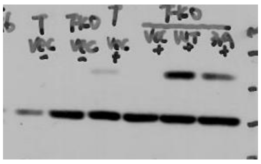

SFB-RAD18 (Chromatin Fraction)

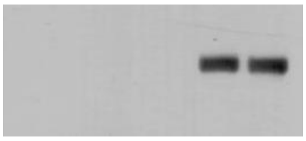

H3 (Chromatin Fraction)

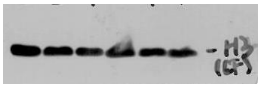

SFB-RAD18 (WCE)

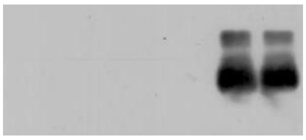

Tubulin (WCE)

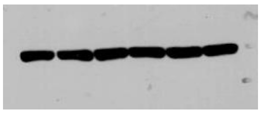

RAD18

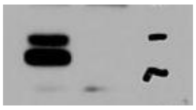

Tubulin

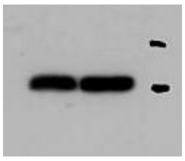

Figure 3E

PCNA (Chromatin Fraction)

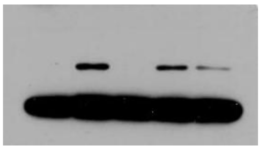

GFP-RAD18 (Chromatin Fraction)

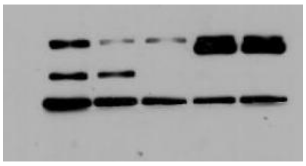

H3 (Chromatin Fraction)

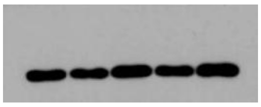

GFP-RAD18 (WCE)

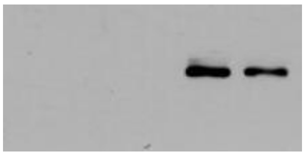

Tubulin (WCE)

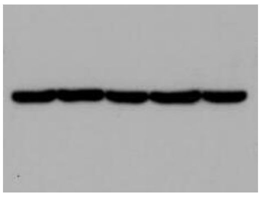

Figure 3F

Ub-PCNA

mUb-PCNA
PCNA

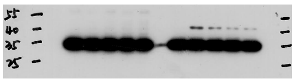

PCNA

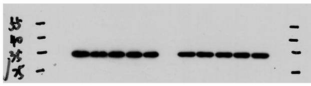

OGT

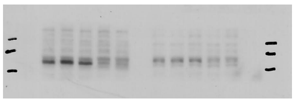

SFB-RAD18

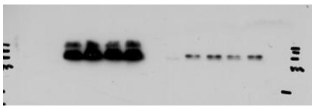

Tubulin

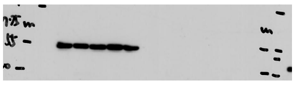

H3

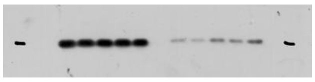

Figure 3G

RAD18

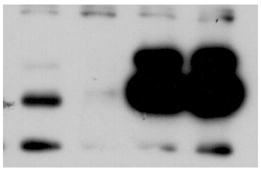

Tubulin

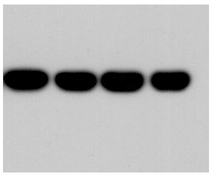

Figure 4A

GFP-Polη

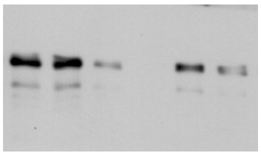

SFB-RAD18

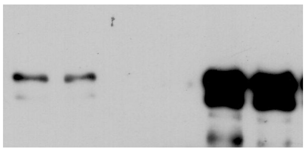

Figure 4B

GFP-Polη

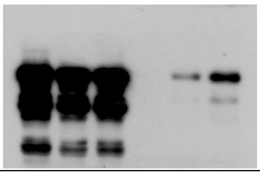

SFB-RAD18

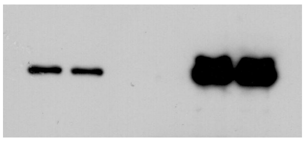

Figure 4C

GFP-Polη

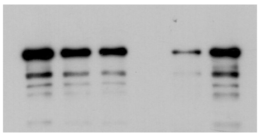

SFB-RAD18

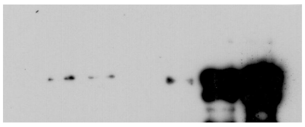

Figure 4D

O-GlcNAc

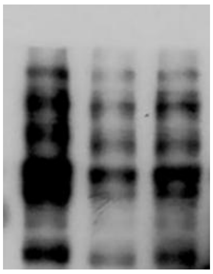

OGT

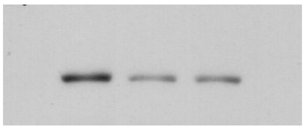

Tubulin

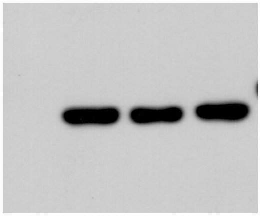

Figure 4E

RAD18-pS434

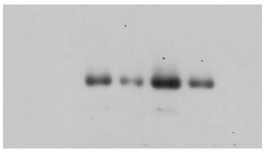

SFB-RAD18 (Input)

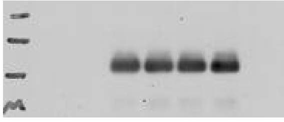

SFB-RAD18 (IP)

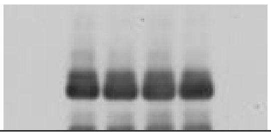

Figure 4F

RAD18

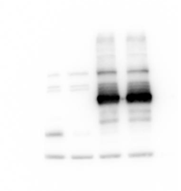

GFP

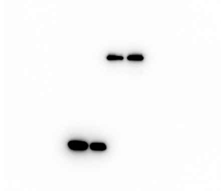

Tubulin

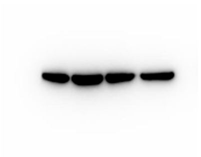

Figure 4G

SFB-RAD18 (IP)

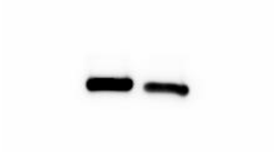

HA-CDC7 (IP)

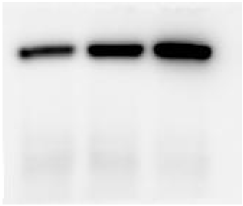

SFB-RAD18 (Input)

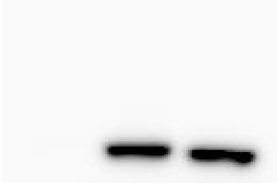

HA-CDC7 (Input)

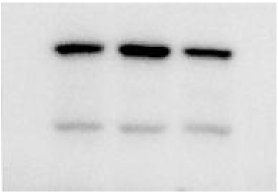

Figure 4H

HA-CDC7

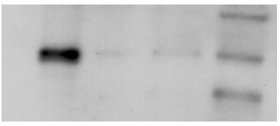

His-RAD18

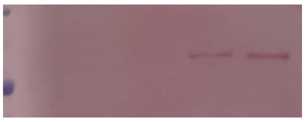

Figure 4I

O-GlcNAc

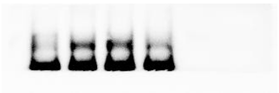

SFB-RAD18 (Input)

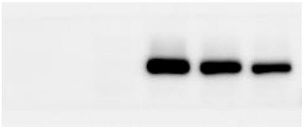

SFB-RAD18 (IP)

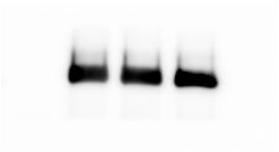

Figure 5A

RAD18

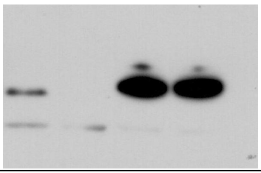

Tubulin

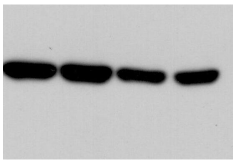

Figure 5D

SFB-RAD18
(Short Exposure)

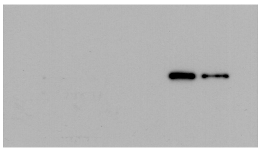

SFB-RAD18
(Long Exposure)

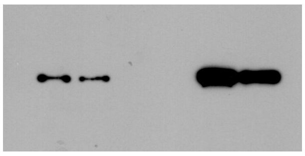

Ponceau S

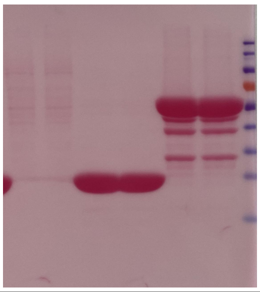

Figure 5E

SFB-RAD51C

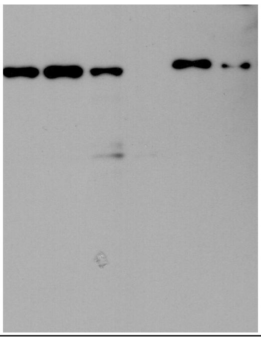

GFP

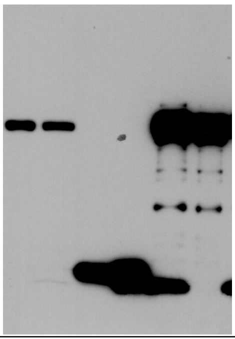

Figure 5F

SFB-RAD18
(Short Exposure)

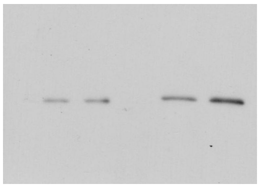

SFB-RAD18
(Long Exposure)

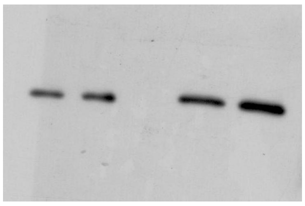

His-RAD51C

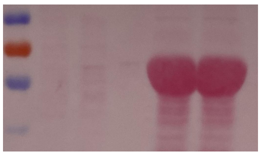

Figure 6B

RAD18

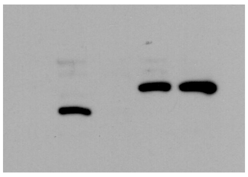

Tubulin

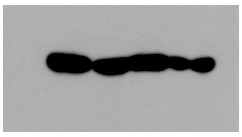

Figure S1A

SFB-RAD18

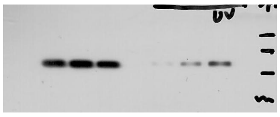

HA-OGT

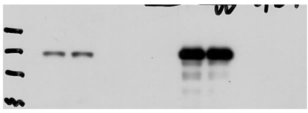

Figure S1B

O-GlcNAc (IP)

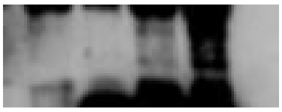

RAD18 (IP)

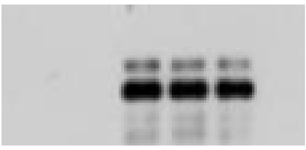

RAD18 (Input)

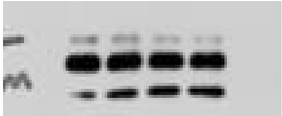

Figure S2A

mUb-PCNA

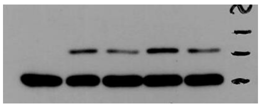

PCNA

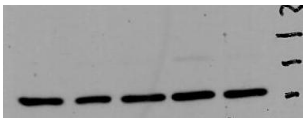

Figure S2B

mUb-PCNA

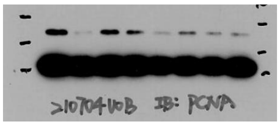

PCNA

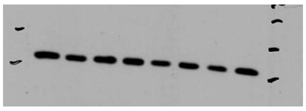

GFP

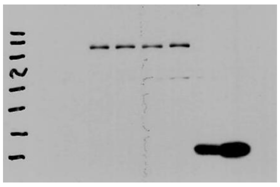

RAD18

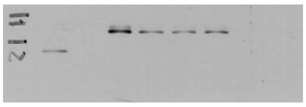

Tubulin

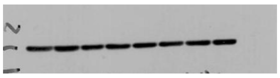

Figure S2C

mUb-PCNA (Short Exposure)

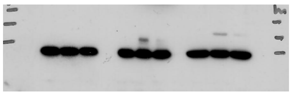

mUb-PCNA (Long Exposure)

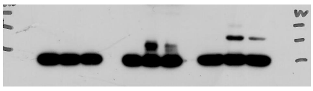

SFB-RAD18 (Short Exposure)

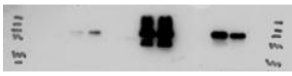

SFB-RAD18 (Long Exposure)

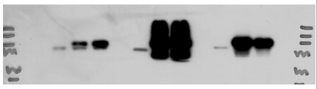

Tubulin

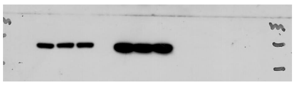

H3

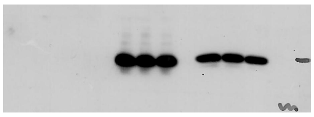

Figure S3A

Flag-RAD6A (IP)

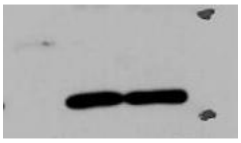

SFB-RAD18 (IP)

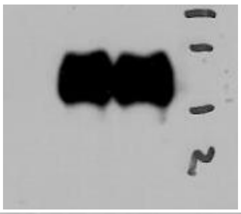

Flag-RAD6A (Input)

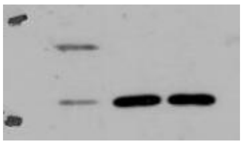

SFB-RAD18 (Input)

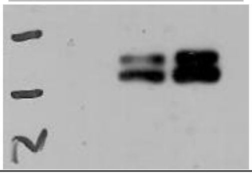

Figure S3B

GFP-RAD6B (IP)

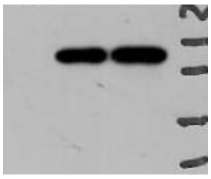

SFB-RAD18 (IP)

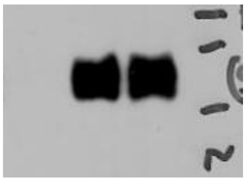

GFP-RAD6B (Input)

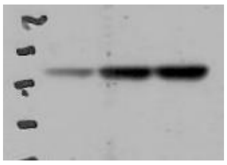

SFB-RAD18 (Input)

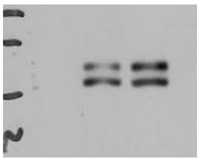

Figure S3C

Flag-Spartan (IP)

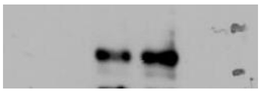

SFB-RAD18 (IP)

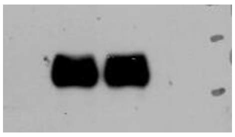

Flag-Spartan (Input)

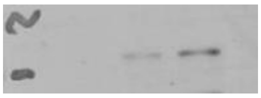

SFB-RAD18 (Input)

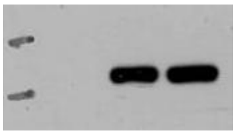

Figure S3D

SFB-SIVA1 (IP)

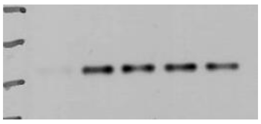

GFP-RAD18 (IP)

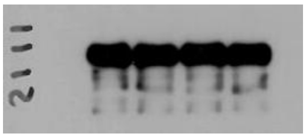

SFB-SIVA1 (Input)

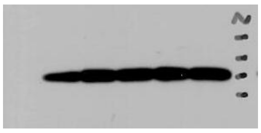

GFP-RAD18 (Input)

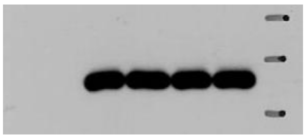

Figure S3E

YFP-NBS1

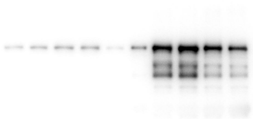

SFB-RAD18

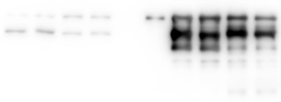

Figure S3F

GFP-Polη

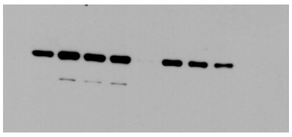

SFB-RAD18

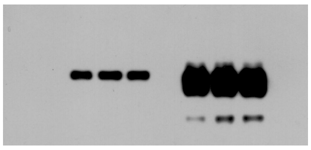

Figure S4A

RAD18-pS434

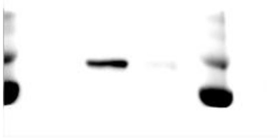

SFB-RAD18 (Input)

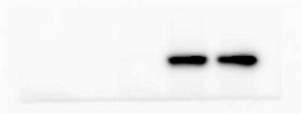

SFB-RAD18 (IP)

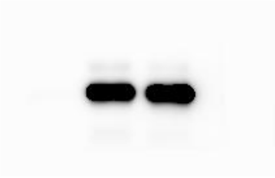

Figure S4B

RAD18-pS434

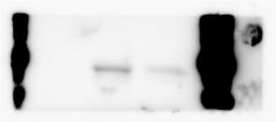

SFB-RAD18 (Input)

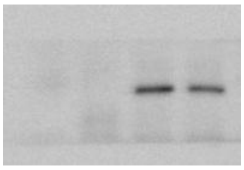

SFB-RAD18 (IP)

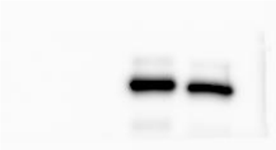

Supplement: Supplementary file 2 — Original Western Blots [file 41419_2024_6700_MOESM2_ESM.pdf]
